# Development of a flow cytometry method to measure antidrug antibodies against CAR T cells

**DOI:** 10.1093/immhor/vlag011

**Published:** 2026-04-25

**Authors:** Georgia Day, Francisco Javier Sánchez-Martín, Lisa Seavers, Karen Cadwallader, Sara Morgado-García

**Affiliations:** Immunology and Immunotoxicology Department, Labcorp, Huntingdon, United Kingdom; Immunology and Immunotoxicology Department, Labcorp, Huntingdon, United Kingdom; Immunology and Immunotoxicology Department, Labcorp, Huntingdon, United Kingdom; Immunology and Immunotoxicology Department, Labcorp, Huntingdon, United Kingdom; Immunology and Immunotoxicology Department, Labcorp, Huntingdon, United Kingdom

**Keywords:** ADA, CAR T cells, cut point, flow cytometry

## Abstract

Chimeric antigen receptor (CAR) T cell therapies are based on the genetic modification of the T cell receptor of a patient’s own T cells. The resulting CAR T cells cytotoxic response is redirected against a specific tumor antigen. This innovative immunotherapy has been used successfully to treat blood malignancies, and it is also being developed as treatment for solid tumors. CAR T cells can lead to immune related adverse outcomes associated with an unwanted immune response in the host, ranging from acute events to those sustained with time, such as antidrug antibody (ADA) development, which can impact the efficacy and persistence of the CAR T cells once administered to the patient. The development of ADAs as response to a drug treatment is not exclusive to CAR T cells, and evidence of their production has been long acknowledged. While traditional types of analysis aim to measure the presence of ADAs by immunoassay methods, with the therapeutic agent being cells, the use of a flow cytometry approach has become the obvious choice for detection and quantification of ADAs against CAR T cells products, as well as any other cell therapies. Here, we present the results of the development of a flow cytometry method to measure ADAs using CAR T cells with an assay to detect the binding of an ADA-like molecule to the CAR T cells in a dose-dependent manner. This method allowed for quantification of ADAs and determination of the assay values needed for potential ADA measurement in clinical samples.

## Introduction

Chimeric antigen receptor (CAR) T cell therapies have been effectively and successfully used since they gained the scientific community’s attention in the late 1990s and early 2000s, after almost miraculously saving young Emily Whitehead’s life, as the first pediatric patient ever to receive this therapy, back in 2012 from her diagnosed and relapsed acute lymphoblastic leukemia.^1^

The use of immunotherapy for cancer treatment started many years ago, with the use of toxins (1890 s, Coley’s toxins)^2^^,^^3^ and cytokines to stimulate the immune system to promote antitumor activity. Over the past 30 years, the field has evolved to more sophisticated treatments from monoclonal antibody–based therapies, including the breakthrough that was the use of immune checkpoint inhibitors, now one of the most important immunotherapies, to adoptive cell therapies, with the use of tumor-infiltrating lymphocytes and CAR T cells.^4^^,^^5^

CAR T cell therapy relies on the use of the patient’s natural T cells genetically modified by the addition of a synthetic chimeric antigen receptor (CAR) that directs the T cells against a tumor antigen. Following this modification, the CAR T cells are cultured and expanded in vitro before being reinfused into the patient as a cancer treatment. These genetically modified CAR T cells effectively identify and eliminate cells expressing the tumor-associated antigen.^6^^,^^7^

Today’s CAR T cell therapies are produced from a patient’s own T cells; however, the development of allogeneic CAR T cells derived from healthy donors can overcome some of the challenges of this therapy, like availability and scalability, or even efficiency and yield of the final product.^8^ Still, this requires the management of graft-versus-host disease, which is the primary risk for toxicity in allogeneic T cell treatment.

CAR T cells can also present safety challenges during their use. This includes the development of adverse events due to rapid immune activation induced by the CAR T cells. Among the most clinically relevant adverse events are cytokine release syndrome (CRS) and immune effector cell–associated neurotoxicity syndrome (ICANS).^9^ Another obstacle to CAR T cell efficacy is the immunogenicity of CAR T cells themselves. The various components of the CAR expressed on the T cell surface may be identified as foreign and elicit an immune response that can result in the production of anti-CAR antibodies, illustrating that the immune response of the host is critical for the CAR T cell therapy success.^10^

The immunogenicity of the CAR T cells has been previously investigated by several authors and has been addressed within discussion platforms like Workshops on Recent Issues in Bioanalysis.^11^ It is now clear that the modifications applied to T cells to manufacture CAR T cells can potentially lead to the development of humoral and cellular immune responses by the host, and these can impair the efficacy and persistence of CAR T cells once infused to the patient.^10^^,^^12^^,^^13^ The immune responses after CAR T cell administration develop mainly due to nonhuman sequences present on the CAR structure, such as domains of murine parental antitarget antibody as well as nongermline sequences found in humanized or fully human antibodies, foreign sequences present in the CAR transgene, or residual viral proteins from the gene editing steps.^10^^,^^12^ Moreover, there are also cases in which the manufacture of CAR T cells involves the use of allogeneic or pseudo-allogeneic cells, with the additional risk of potential development of graft-versus-host disease.^14^

The characterization of the host immune response is key to the success of the CAR T cell therapy. The presence of humoral responses such as antidrug antibodies (ADAs) has been previously studied and characterized using different analytical approaches (antigen binding tests, electrochemiluminescence assays, radioimmunoassays, enzyme-linked immunosorbent assay, quantitative polymerase chain reaction, flow cytometry).^12^^,^^15^ Regulatory agencies like the Food and Drug Administration (FDA) recommend developing assays to detect humoral and/or cellular immune responses against the CAR T cells.^16^

The development of an assay able to detect and monitor the presence of ADAs against the CAR T cells can help to characterize the immune response as well as the CAR T product before administration to the patients, as well as during and ongoing treatment. Several advantages have been described on the use of flow cytometry to measure humoral response against CAR T cells, mainly related to the CAR expressed on the T cells surface in its native state, preventing processing steps that could mask some epitopes.^11^

Here, we present our approach to an ADA detection and quantification assay developed by flow cytometry in line with similar publications.^17^^,^^18^ This flow cytometry method has already been successfully used at Labcorp to support ADA detection for different CAR T cell products.

## Methods

### Cell culture of naïve and CAR T cells, and Raji cells

ROR1 is a membrane receptor that plays a key role in development. It is highly expressed during the embryonic stage and is involved in cell differentiation, which leads to the formation of functional tissues and organs.^19^^,^^20^

In adult tissues the expression of ROR1 is downregulated, being almost absent in some tissues. This downregulation is important, as the overexpression in adult tissues is associated with pathological conditions. Malignancies such as leukemias (B cell acute lymphoblastic leukemia [B-ALL], chronic lymphocytic leukemia [CLL]), which is the most studied in the context of ROR1 expression and targeted malignancy; lymphomas (mantle cell lymphoma [MCL], diffuse large B cell lymphoma [DLBCL]); and some solid tumors overexpress ROR1, making it a promising target for cancer treatment.^19^^,^^20^

As such, monoclonal antibodies, small molecules, and bispecific antibodies and bispecific T cell engagers targeting ROR1 have been used in cancer therapies.^21^ CAR T cell therapies targeting ROR1 are also gaining interest in potential therapeutics to tumors that overexpress ROR1, such as leukemias, lymphomas, and solid tumors.^19–21^

Raji is the first continuous human cell line of hematopoietic origin and was derived in 1964 from a Nigerian patient with Burkitt’s lymphoma.^22–24^ These cells have become a powerful research tool and often, used as targets in CAR T cells models both in vitro and in vivo.

Naïve and ROR1 CAR T cells (CD4 and CD8), and Raji cells expressing ROR1 antigen and green fluorescent protein (GFP), were generated, and kindly provided by the Universitätsklinikum Würzburg team within imSAVAR (Immune Safety Avatar) consortium (https://imsavar.eu/). Labcorp is a contributing partner of imSAVAR, which aims to improve the efficacy and safety of immunomodulatory therapies.

Naïve and ROR1 CAR T cells (CD4 and CD8) derived from the same donor were cultured in RPMI 1640 (Gibco) supplemented with 10% commercial human AB serum (Sigma-Aldrich), 1% penicillin-streptomycin (Gibco), 0.1% 2-mercaptoethanol (Gibco), and 50 u/mL IL-2 (Miltenyi Biotec). Cells were maintained at 37 °C and 5% CO_2_ at a cell density not lower than 1 × 10^6^ cells/mL.

Raji cells expressing ROR1 were cultured in RPMI 1640 (Gibco) supplemented with 10% heat-inactivated fetal bovine serum (Gibco) and 1% penicillin-streptomycin (Gibco). Cells were maintained at 37 °C and 5% CO_2_ at a cell density between 0.5 and 1 × 10^6^ cells/mL.

### ADA proof-of-concept assay by flow cytometry

Briefly, ROR1 CAR T cells (CD4 and CD8) were incubated with increasing concentrations (0–100 µg/mL) of ROR1-immunoglobulin (IgG)_1_, a protein chosen to act as an ADA-like molecule (Recombinant Human ROR1 Fc Chimera Protein; R&D Systems) in the presence of 1 in 10 diluted commercial human AB serum (Sigma-Aldrich). An isotype (Recombinant Human IgG_1_ Fc; R&D Systems) was used as negative control (Fig. S1). The binding was detected using an anti-human IgG + IgM PE antibody [R-Phycoerythrin AffiniPure F(ab′)_2_ Fragment Goat Anti-Human IgG + IgM (H + L); Jackson ImmunoResearch]. The median fluorescence intensity (MFI) of the IgG + IgM PE detection antibody was used as reference value to determine the binding of the ADA-like antibody. Naïve CD4 and CD8 cells were also included in the assay as negative controls. Naïve cells were only incubated with the highest concentration of ROR1-IgG_1_ (100 µg/mL) and isotype (Recombinant Human IgG_1_ Fc).

**Figure 1:**
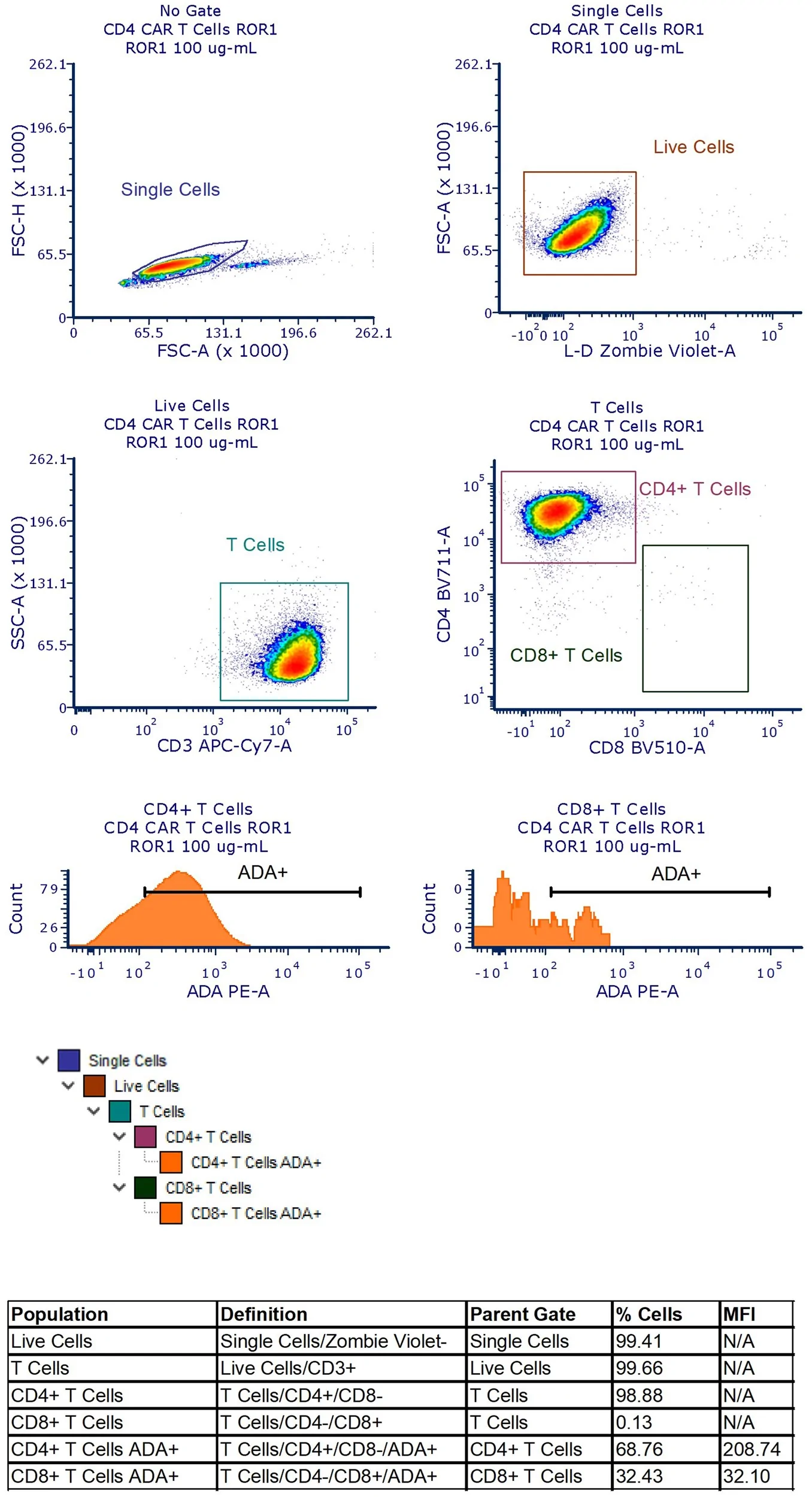
Naïve or ROR1 CD4 CAR T cells were defined following the sequential gating strategy shown. Briefly, cells were gated to exclude doublets, followed by an exclusion marker for dead cells. After selecting the live cells, CD4 T cells were defined by the positive expression of the CD3 marker as well as the expression of CD4 and the absence of CD8. The binding of the ADA was then measured in all CD4 T cells in a histogram using the MFI value of the detection antibody. FSC-A, forward scatter area; FSC-H, forward scatter height; L-D, live/dead; N/A, Not Applicable; SSC-A, side scatter area.

**Figure 2:**
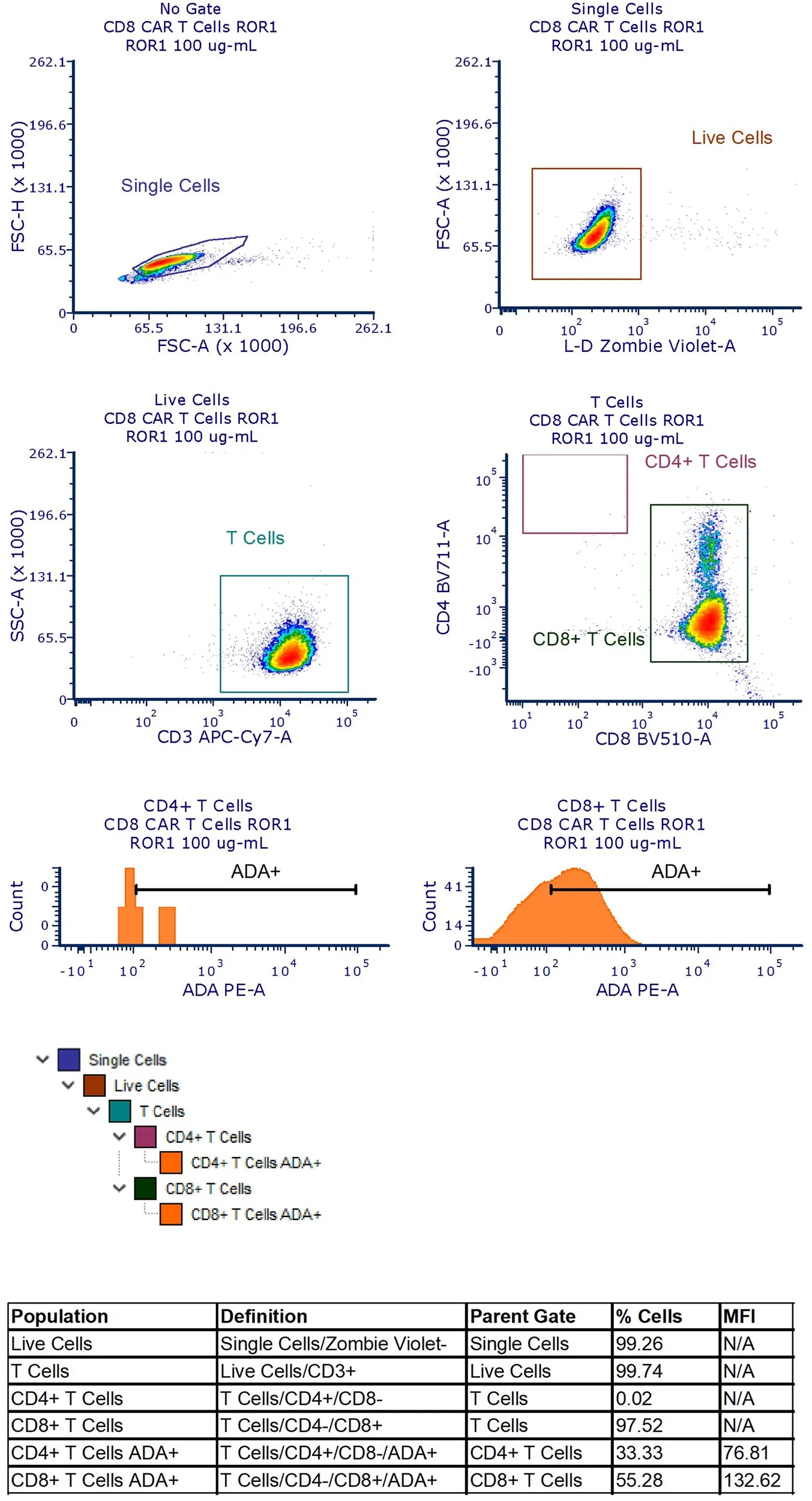
Naïve or ROR1 CD8 CAR T cells were defined following the sequential gating strategy shown. Briefly, cells were gated to exclude doublets, followed by an exclusion marker for dead cells. After selecting the live cells, CD8 T cells were defined by the positive expression of the CD3 marker as well as the expression of CD8 and the absence of CD4. The binding of the ADA was then measured in all CD8 T cells in a histogram using the MFI value of the detection antibody. FSC-A, forward scatter area; FSC-H, forward scatter height; L-D, live/dead; N/A, Not Applicable; SSC-A, side scatter area.

A live/dead dye (Zombie Violet; BioLegend) and cell surface lineage markers (CD3 APC-Cy7, CD4 BV711, CD8 BV510; BD Biosciences) were also included in the CAR T cell characterization flow panel. Briefly, cells were gated to exclude doublets, followed by the exclusion marker for dead cells. After selecting live cells, CD4 and CD8 T cells were defined by the positive expression of the CD3 marker as well as the presence or absence of CD4 and CD8 expression (CD4^+^CD8^−^ and CD4^−^/CD8^+^, respectively). ADA-like molecule binding was measured in CD4 and CD8 T cells in a histogram using the MFI value of the detection antibody (Figs. 1 and 2).

Samples were acquired on a BD FACSLyric flow cytometer and data was analyzed with FCS Express (V6.03.0011; DeNovo Software).

### Cytotoxicity assay

**Figure 3:**
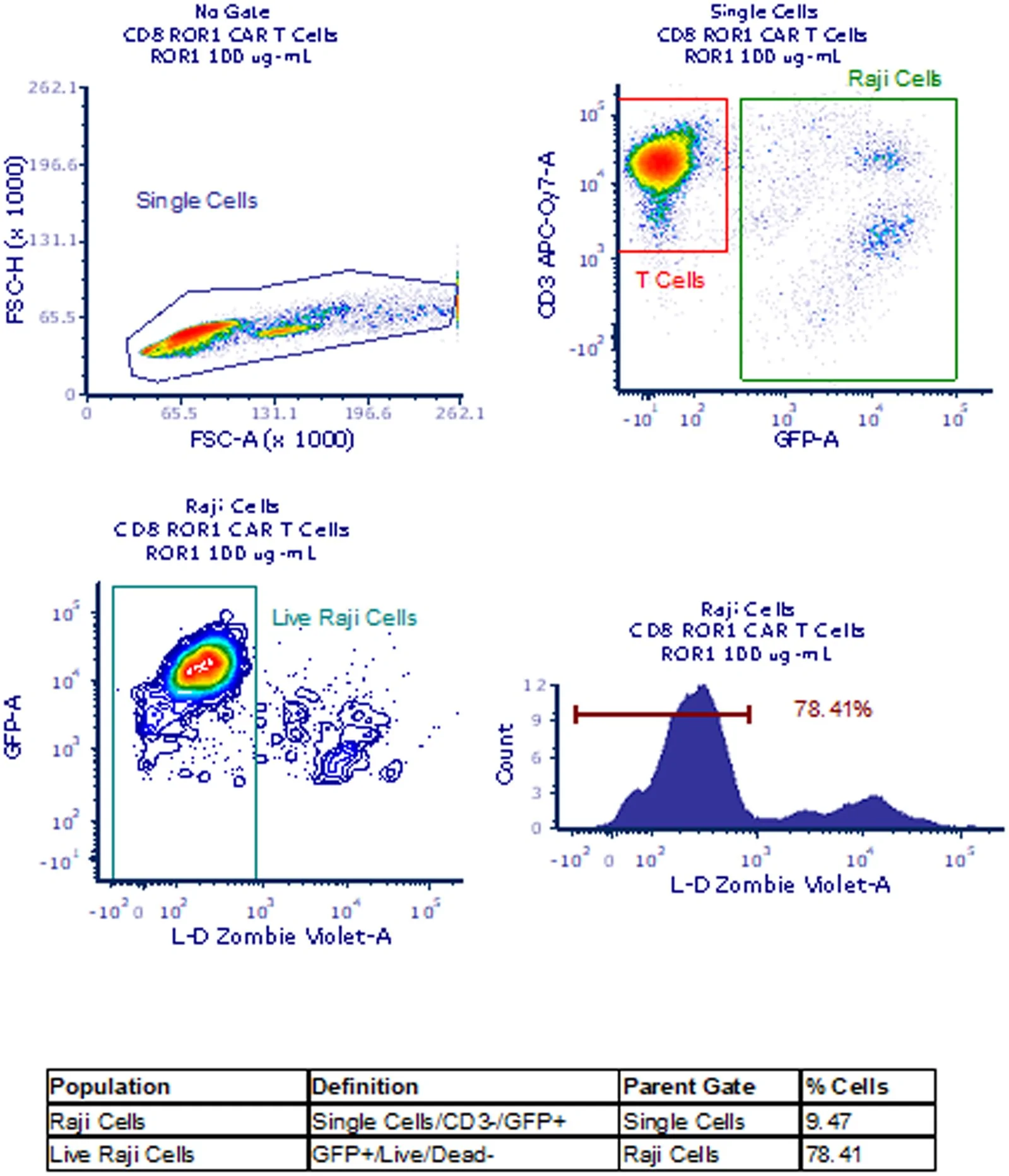
The cytotoxicity assay of CAR T cells and Raji cells was analyzed by flow cytometry following the gating strategy shown. Briefly, all cells were gated and subsequentially defined as T cells or Raji cells by the expression/absence of CD3 and GFP (T cells CD3^+^ GFP^−^; Raji cells CD3^−^ GFP^+^). The percentage of live Raji cells was measured using a live/dead (L-D) dye. FSC-A, forward scatter area; FSC-H, forward scatter height.

The cytotoxic activity of the CAR T cells was tested in a killing assay. Effector ROR1 CAR T (CD4 and CD8) cells were incubated with a target cell line (Raji cells expressing ROR1 and GFP), in the presence or absence of ROR1-IgG_1_ (ADA-like molecule) to assess the interference of this ADA-like molecule with the cytotoxicity of the CAR T cells. Briefly, ROR1 CAR T cells were preincubated with ROR1-IgG_1_ for 30 min before coculture with the target cell line Raji at different effector-to-target (E:T) ratios (1:10, 1:1, 10:1; 1 = 25,000 cells) for 16 to 24 h. Cocultured cells were then stained with CD3, and CD4 or CD8 to determine effector cells. Briefly, all cells were gated and subsequentially defined as T cells or Raji cells by the expression/absence of CD3 and GFP (T cells, CD3^+^ GFP^−^; Raji cells, CD3^−^ GFP^+^) (Fig. 3). The percentage of live Raji cells was measured using a live/dead dye (Zombie Violet; BioLegend).

### Cytokine measure

Supernatants from cytotoxicity assays were analyzed for the presence of inflammatory cytokines using a multiplex immunoassay (FirePlex Human Inflammation-Immunoassay Panel; Abcam). This predesigned kit was chosen as an option for fast and easy quantification of key cytokines and inflammatory markers. Samples were analyzed following manufacturer’s recommendations for quantification of IFN-γ, IL-1β, IL-6, IL-8, IL-10, IL-12p70, MCP1, and TNF-α. Data were analyzed with FirePlex Analysis Workbench (V2.0.274; Abcam).

### Cut point calculation

The cut point is defined as the level of response at and above which a sample is defined to be a “reactive” (often called “potential positive”) for the presence of ADA, and below which it is probably negative.^25^ To define the level of matrix background signal, a plate-specific cut point was calculated by incubating naïve and CAR CD8 T cells with serum diluted 1 in 10 from 16 naïve donors obtained from the Labcorp internal donor program. The human blood collection policy at Labcorp adheres to the Human Tissue Act 2004. Background binding was then detected by flow cytometry with the same anti-human IgG + IgM PE antibody used to detect the ADA-like molecule ROR1-IgG_1_. The MFI values from the IgG + IgM PE obtained from naïve and ROR1 CAR CD8 T cells were used to calculate the cut point for the assay. After the evaluation and removal of analytical and biological outliers, the cut point on normalized signal responses was determined based on the calculation of mean signal plus 3.09 times the standard deviation (SD) (cut point = mean + 3.09 × SD).^26^^,^^27^ A fixed correction factor of 3.09 is commonly applied to establish a high-stringency cut point. This value corresponds to the 99.9th percentile of a standard normal distribution, ensuring a false positive rate of ≤0.1%. The approach is statistically justified and widely accepted in regulatory guidance to maintain assay specificity.^25^^,^^28^

## Results

### ADA-like molecule binds to CAR T cells in a dose-dependent manner

A chimeric molecule, ROR1-IgG_1_, was chosen to act as the ADA molecule in this system. ROR1-IgG_1_ was incubated with either CD4 or CD8 ROR1 CAR T cells, and the binding was then identified using a detection antibody against human IgG + IgM.

**Figure 4:**
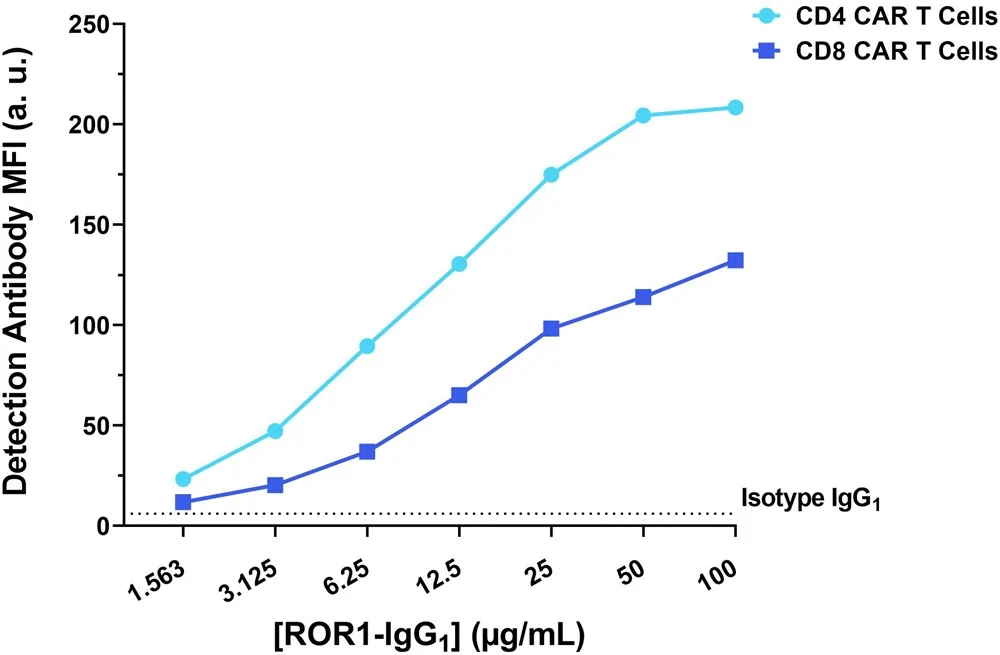
The ROR1-IgG_1_ chimeric protein bound to ROR1 CAR T cells (CD4 and CD8) in a dose-dependent manner. Binding was measured by flow cytometry using a PE conjugated antibody for detection of hIgG_1_. Shown are data representative from 1 experimental occasion (total number of experimental occasions: n = 3). a.u., arbitrary units.

Results showed that the ADA-like molecule bound to the ROR1 CAR T cells (both CD4 and CD8) in a dose-dependent manner (Fig. 4). CD4 ROR1 CAR T cells showed a higher MFI than CD8 ROR1 CAR T cells; however, a similar binding pattern was observed for both cell lines.

Naïve CD4 and CD8 T cells were incubated with the highest concentration of ROR1-IgG_1_ (100 µg/mL) and isotype (Recombinant Human IgG_1_ Fc). No binding of the ADA-like molecule was detected in these cells (data not shown). ROR1 CAR T cells (both CD4 and CD8) were also incubated with the isotype IgG_1_ Fc (100 µg/mL), and the binding was used as a negative control for the analysis of the ROR1-IgG_1_ binding (ADA^+^) (Fig. S1).

### ADA-like molecule blocks cytotoxicity of CD8 CAR T cells in a dose-dependent manner

**Figure 5:**
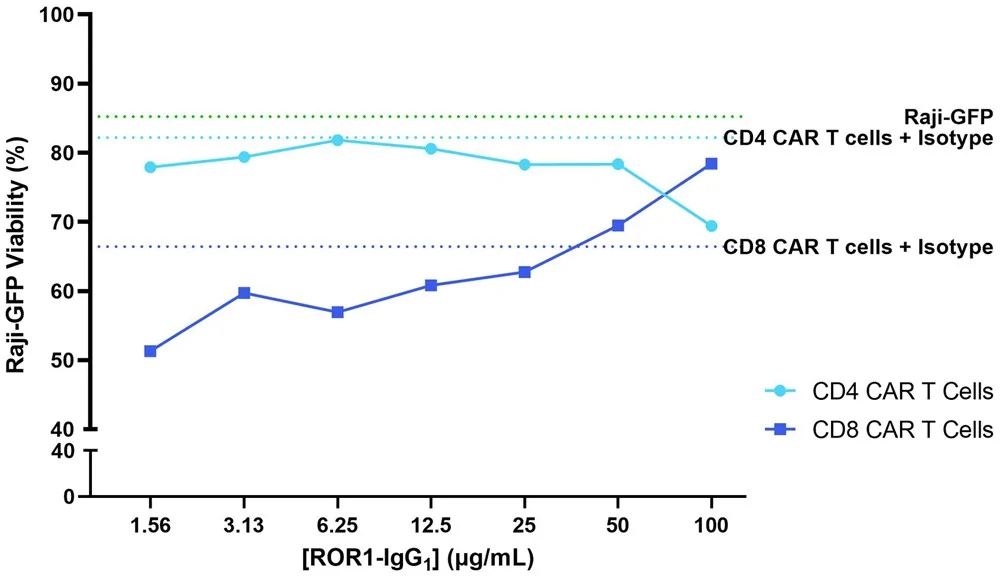
Cytotoxic activity of ROR1 CD8 CAR T cells was blocked by ROR1-IgG_1,_ an ADA-like molecule in a dose-dependent manner. Raji cells expressing ROR1 were killed by ROR1 CD8 CAR T cells and this cytotoxic activity was impaired by the presence of the ADA-like molecule ROR1-IgG_1_. ROR1 CD4 CAR T cells did not show any cytotoxicity against target cell line Raji. Shown are data representative from 1 experimental occasion (total number of experimental occasions: n = 2).

**Figure 6:**
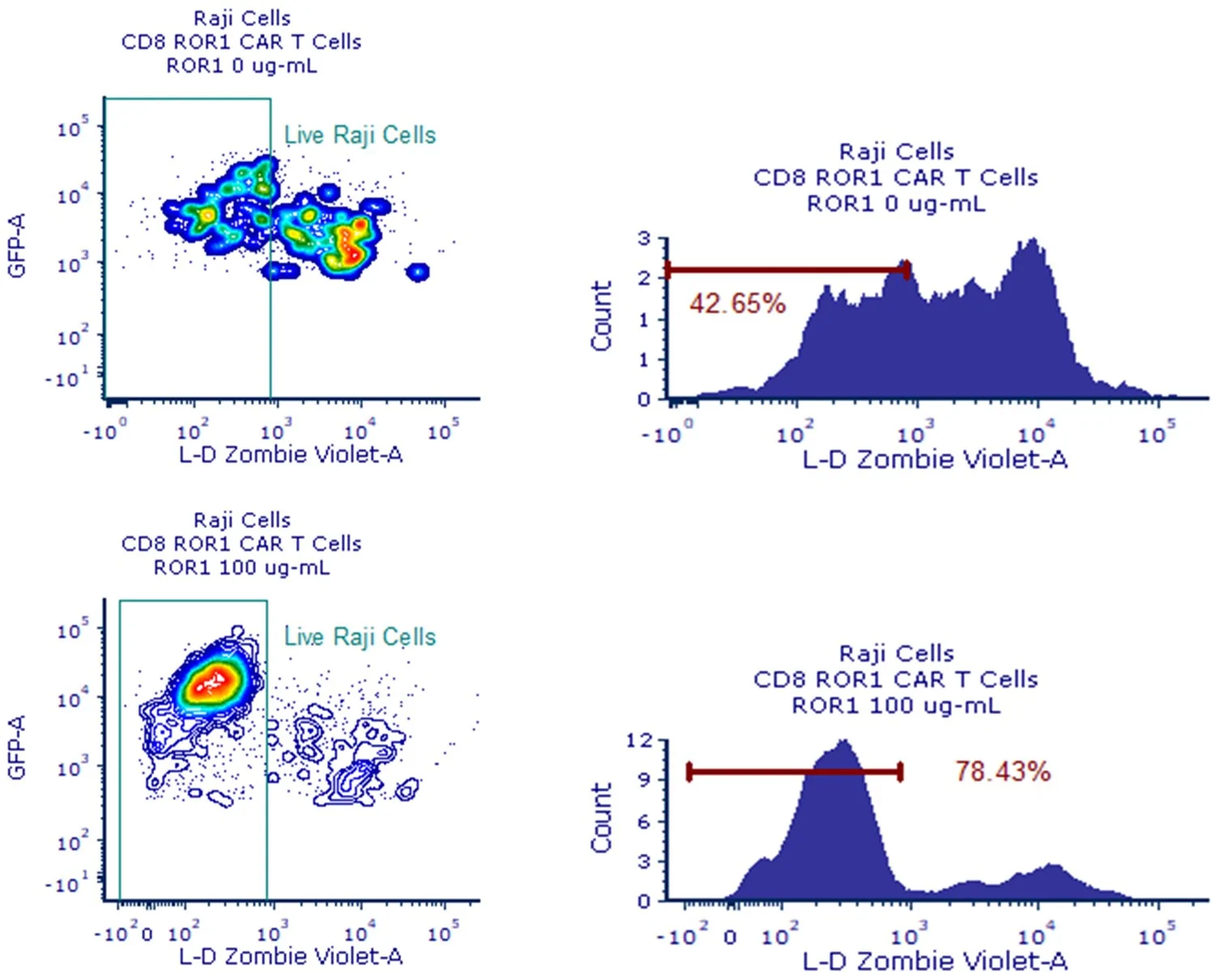
Representative flow cytometry images showing cytotoxic activity of ROR1 CD8 CAR T cells against Raji cells expressing ROR1 (E:T ratio  = 10:1) (top). Cytotoxicity was blocked by ROR1-IgG_1_ ADA-like molecule (bottom). Shown are data representative from 1 experimental occasion (total number of experimental occasions: n = 2). L-D, live/dead.

The functionality of the CAR T cells and interference of the ADA-like molecule was assessed in a killing assay using Raji cells expressing the CAR target molecule ROR1 as targets. Cytotoxic activity was analyzed by flow cytometry, measuring the viability of Raji-GFP cells after the overnight coculture. Although 3 different E:T ratios were assessed, only the 10:1 E:T ratio showed reliable results regarding cytotoxicity (data from 1:1 and 1:10 E:T ratios are shown in Fig. S2). CD8 CAR T cells showed cytotoxic activity against the Raji target cells, and this cytotoxicity decreased in a dose-dependent manner when effector cells were incubated with the ADA-like molecule ROR1-IgG_1_ before the coculture (R^2^ = 0.77) (Fig. 5, dark blue line). This blocking activity was dose dependent, with near 50% increase in Raji cells viability (from 42.65% up to 78.43%) when effector cells were incubated with the highest concentration of ROR1-IgG_1_ (100 µg/mL) (Fig. 6).

Contrarily, CD4 ROR1 CAR T cells did not show any cytotoxic activity, and the preincubation of CD4 ROR1 CAR T cells with the ROR1-IgG_1_ molecule did not have any impact on the viability of the target cells Raji (Fig. 5, light blue line). These results could be anticipated, as CD4 T cells were not expected to directly kill the target cells, but rather to mainly exert function by the production of cytokines.

### Cytokine measure

Supernatants, from the cocultures (E:T ratio = 10:1) to assess cytotoxicity and the interference of the ADA-like molecule in the CAR T cell function (described in the above section), were assessed for the presence of cytokines using a multiplex kit for human inflammation. Among the 8 cytokines included in the kit, IL-1β and IL-12p70 could not be quantified, as the signal values were too low (below assay limit of detection; data not shown).

Within the cytokines measured, the levels detected for IL-6 were the lowest (between 1 and 9 pg/mL) with the lowermost values corresponding to CAR CD8 T cells (ranging from 1.5 to 3.5 pg/mL), while the highest values were found from CAR CD4 T cells (peak value 9.12 pg/mL). The IL-6 produced by CAR T cells (CD4 and CD8) remained unchanged in the presence of different concentrations of ROR1-IgG_1_ protein, except for the highest concentration of the ADA-like molecule (100 µg/mL), which decreased IL-6 levels produced by CAR CD4 T cells by approximately 60% (from 8.5 to 3.5 pg/mL), supporting the fact that the ADA-like molecule binding to CAR T cells can impair their functional activity (Fig. 4, Panel A).

**Figure 7:**
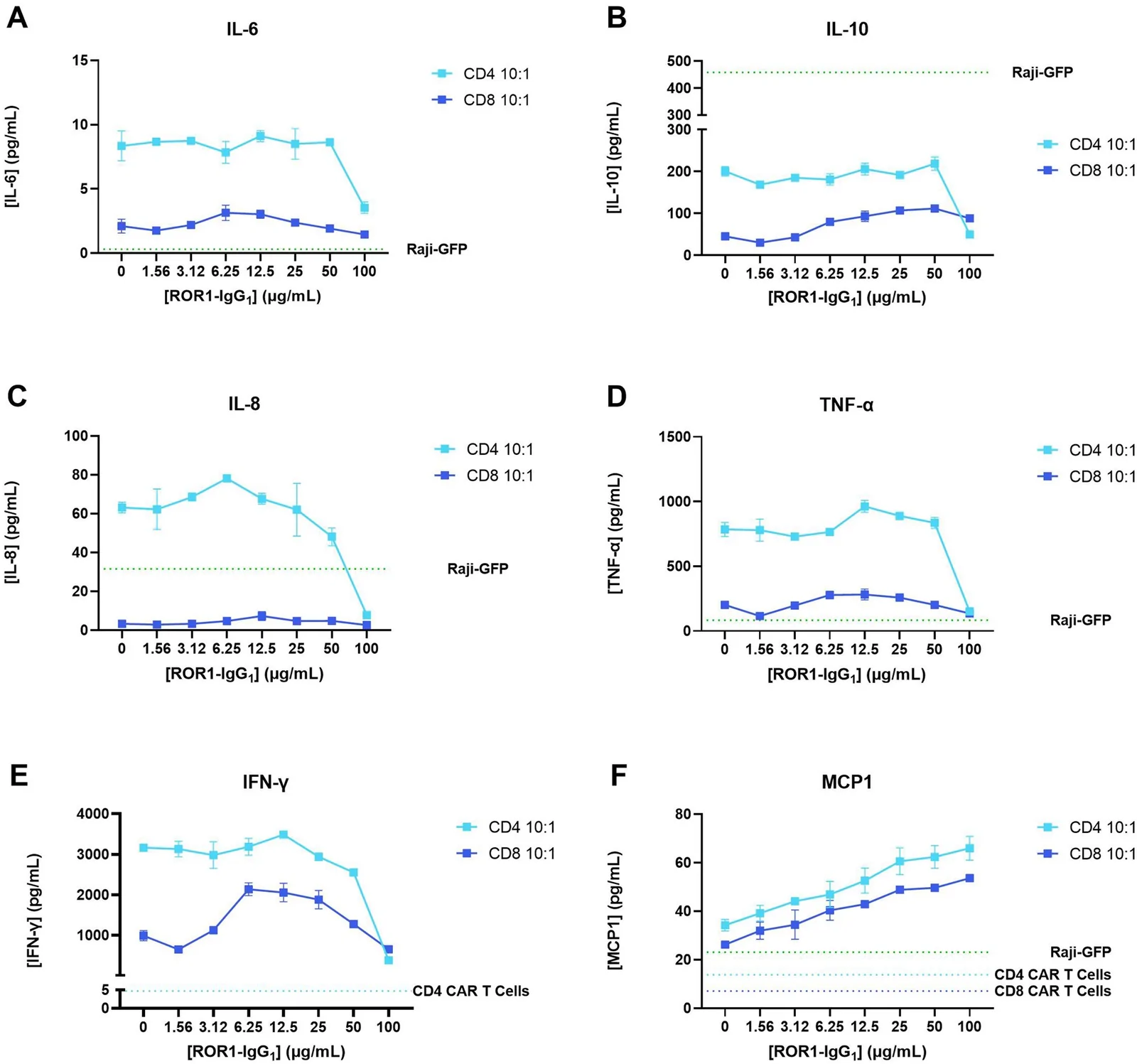
**(A–F)** Cytokine measure (IL-6, IL-10, IL-8, TNF-α, IFN-γ, and MCP1) in supernatants from cocultures of ROR1 CAR T cells (CD4 and CD8) with Raji cells expressing ROR1 (E:T ratio  = 10:1). Shown are mean ± SD of 2 replicates (total number of experimental occasions: n = 1).

This was also the case for IL-10, with higher concentrations detected for CAR CD4 T cells compared with CAR CD8 T cells. Similarly, a decrease in IL-10 was observed for the highest concentration of the ADA-like molecule, which decreased IL-10 levels from CAR CD4 T cells by over 75% (from 210 to 50 pg/mL). The opposite trend was seen for CAR CD8 T cells, in which higher concentrations of ROR1-IgG_1_ slightly increased the production of IL-10 by these cells in a dose-dependent manner (Fig. 7B). Interestingly, the control of Raji cells alone showed the highest concentration of IL-10 detected.

A similar pattern was again observed for IL-8 and TNF-α for CAR CD4 T cells. These cells were responsible for the production of high levels of these cytokines (maximum detected of 78 and 962 pg/mL for IL-8 and TNF-α, respectively), compared with CAR CD8 T cells, which showed IL-8 production of <10 pg/mL, and TNF-α levels 4 to 5 times lower than that of CAR CD4 T cells (lowest of 115 pg/mL). The concentrations measured for IL-8 remained practically unchanged for CAR CD8 T cells regardless of the concentration of ROR1-IgG_1_ molecule, and the levels of TNF-α slightly increased and then decreased with higher concentration of the ADA-like molecule. However, for CAR CD4 T cells a dose-dependent decrease could be observed when cells were preincubated with high levels of ADA-like molecule (from 12.5 to 100 µg/mL) (Fig. 7C, D).

For IFN-γ, very high concentrations were measured for CAR CD4 T cells (maximum of 3484 pg/mL) and CAR CD8 T cells (maximum of 2136 pg/mL measured). There was a dose-dependent decreasing trend for IFN-γ levels when the effector CAR T cells were preincubated with the ADA-like molecule (from 12.5 to 100 µg/mL). Interestingly, the concentration of IFN-γ remained invariable for CAR CD4 T cells when low or no inhibitory molecule was present; however, the values of IFN-γ produced by CAR CD8 T cells increased with lower concentrations of ROR1-IgG_1_, and then decreased (Fig. 7E). These lower but slightly increasing IFN-γ values detected in the presence of low concentrations of ADA-like molecule could be due to CAR CD8 T cells’ attempt to overcome the inhibitory signal. This same pattern was observed for IL-8 and TNF-α for CAR CD4 T cells, in which small increases were detected at 6.25 and 12.5 µg/mL, respectively, followed by a decrease.

Finally, the levels of MCP1 measured clearly followed a dose-dependent pattern of increase for both types of CAR T cells, with CD4 T cells showing slightly higher concentrations for this cytokine (Fig. 7F).

### Plate-specific cut point calculation with serum from naïve donors

To calculate a plate-specific cut point (defining a value at and above which a sample is considered to be potential positive for the presence of ADA, and below which it is probably negative),^22^ naïve and ROR1 CD8 CAR T cells were incubated with 16 human naïve serum samples diluted 1 in 10 to analyze the background signal coming from the matrix of the general population. Results showed that the ROR1 CD8 CAR T cells had a higher background signal compared with the naïve CD8 T cells. For the calculation of the cut point, the difference in the signal was determined by subtracting the MFI values of the naïve cells from the CAR T cells. The average of the background was calculated from 12 donors (average MFI = 35.33), and the plate-specific cut point was estimated using a correction factor of 0.1% calculated as 3.09 times the SD (cut point = average MFI + SD × 3.09: cut point = 35.33 + 8.96 × 3.09). Therefore, the plate-specific cut point calculated from 12 naïve donors was 63.01 (MFI value) (Table 1).

**Table 1:** Cut point calculation.

Donor ID (MRD 1:10)	ROR1 CAR T cells: CD8^+^ T cells ADA^+^ (MFI)	Naïve T cells: CD8^+^ T cells ADA^+^ (MFI)	Difference (MFI)
Donor 2	51.38	6.32	45.06
Donor 4	51.28	11.89	39.39
Donor 6	75.83	35.00	40.83
Donor 7	50.74	12.51	38.23
Donor 8	40.14	10.70	29.44
Donor 9	60.39	11.80	48.59
Donor 10	49.51	4.21	45.30
Donor 11	51.69	15.52	36.17
Donor 12	33.12	10.85	22.27
Donor 13	38.79	12.56	26.23
Donor 14	37.47	10.13	27.34
Donor 15	33.14	8.07	25.07
		Average	**35.33**
		SD	8.96
		Correction factor - 0.1% (SD*3.09)	27.68
		**Cut point**	**63.01**

MFI values of binding background signal of anti-IgG + IgM PE detection antibody measured on ROR1 CD8 CAR T cells and naïve CD8 T cells incubated with serum from 16 naïve human donors. After the evaluation and removal of analytical and biological outliers, the cut point was determined based on the calculation of mean signal plus 3.09 times the SD (correction factor of 0.1%). Ultimately, the average was 35.33, the SD was 8.96, the correction factor was 27.6, and the cut point was 63.01.

Abbreviation: MRD, Minimum Required Dilution.

Extrapolating these results to the binding of the ADA-like molecule to ROR1 CD8 CAR T cells (Fig. 4), a concentration of ADA of 12.5 µg/mL (measured as MFI = 65.11) would be the sensitivity of the assay. This value would be the threshold to determine the positivity or negativity for the presence of ADAs in a sample. The obtained sensitivity allows to define the levels for low and high positive controls, moving further into outlining a robust assay.

## Discussion

The modification or stimulation of a patient’s immune system to treat cancer by directing or modulating the immune response against the tumor has been a major breakthrough, changing the way cancer is treated, and opening new possibilities in the field. These therapies have taken time to define and refine and are in continuous development to reach even the most challenging types of cancer.

CAR T cell therapy takes the patient T cells and modifies them to express a chimeric T cell receptor that redirects the T cell immune response against a tumor antigen. These therapies have been very successful up to date, with 6 CAR T products approved by the FDA and 4 of them also approved by the European Medicines Agency. These CAR T cells target CD19 or B cell maturation antigen to treat hematological malignancies.^29^ New targets are being tested and developed constantly, including attempts for treating solid tumors.^30^ The progressive improvement of the CAR T cells and their combined use with immunomodulatory drugs have increased the complexity and success of these therapies.^31–33^

Allogeneic CAR T therapies go a step further and aim to manufacture T cells off the shelf, ready to be administered to appropriate patients. This would eliminate some of the hurdles presented by CAR T cell therapy, like yield, time constraints, and availability, providing cells ready to use for treatment.^8^^,^^29^^,^^30^ Furthermore, the availability of allogenic CAR T cells would help expedite the development of assays to characterize the potential immune response, including ADA production.

Despite the simplicity of the idea of using the immune system for cancer treatment, immunotherapy requires careful planning and intensive initial follow-up to ensure the patient’s response is adequate, and to rapidly mitigate any potential exacerbated immune-related toxicity.^10^^,^^12^^,^^13^

Unwanted immune responses have been observed after infusion of CAR T cell products to patients, and although CRS and ICANs are relatively well-characterized adverse events, this highlights the need for continuous immunogenicity definition and handling, to define the safety of the CAR T cells administered to patients.^9^^,^^34^^,^^35^ The FDA regulatory guidelines for the development of CAR T cell products includes immunogenicity as part of the clinical pharmacology considerations to monitor this therapy, given the potential impact of immunogenicity on clinical outcomes.^17^

The development of ADAs against a therapeutic is a well-known response and has been extensively studied and characterized, due to the adverse events that ADAs can potentially induce. This includes ADAs altering efficacy by affecting clearance, pharmacodynamics, and pharmacokinetics, or even completely neutralizing the drug^36^ to more severe infusion reactions caused by hypersensitivity.^37^

Vaisman-Mentesh et al.^38^ reviewed the ADA response after treatment with monoclonal antibodies, and while the molecular mechanisms of ADA generation are not fully understood, it is known to be dependent on both patient and drug characteristics. This concept can be applied to any therapeutic, including CAR T cells, as these two elements will determine the potential host immune response. In this sense, both cellular and humoral responses against CAR T cells have been described and their impact has been studied.^10^^,^^12^^,^^13^^,^^39^

The measurement and monitoring of an ADA response against CAR T cells is important to determine the success of the treatment. While the presence of ADAs may not be life-threatening compared with acute responses like CRS or ICANs, monitoring this immune response remains critical to evaluate the persistence of CAR T cells after infusion. It has been generally accepted that the success of CAR T treatment is highly dependent on the persistence of CAR T cells in circulation, and this can be affected by the presence of an ADA response.^12^ Low levels of binding antibodies for a short period of treatment may not have clinical relevance, but a persisting high level of ADAs can lead to a complete loss of response.^38^

ADA responses have been qualitatively measured using different immunoassay methods, mostly based on the high specificity of antibodies binding their targets. These methods are accepted by the regulatory bodies and are normally structured in a 3-tiered approach, consisting in a first screening assay, followed by a confirmatory, and final titration and neutralization assays.^28^

Naturally, in the case of CAR T cell products, the treatment agent is the cells, and to measure antibody production against these cells after administration to the patients, new approaches have been developed and are currently in use. The use of flow cytometry is strongly supported by the fact that any analyses are performed on the unchanged product (i.e. CAR is expressed in the cells in its functional form and any in vivo interactions between surface molecules, or cell to cell, can still be considered).

Current guidelines encourage an extensive characterization of the CAR T cell product, typically done by flow cytometry. These assessments include cell viability, identity, purity, strength, and CAR detection to determine the percentage of CAR-positive cells.^16^ Functional assays to corroborate the cytotoxic activity of CAR T cells are also performed as part of the initial characterization of the cells, and some of these assays include endpoints that can also be measured by flow cytometry.^40^ Several studies have already been published establishing the use of flow cytometry assays not only to characterize the CAR T cell product,^41^ but also to monitor the immune response and the CAR T cell persistence after infusion,^40^^,^^42–45^ and to detect ADA development against the CAR T cells.^45^ Some of these flow cytometry approaches showed validated methods^46^ ensuring the robustness and reproducibility of the assay, and new panels are being developed with more endpoints thanks to the increased capabilities of spectral flow cytometry.^47–51^

## Conclusion

In this work, we present the development of a flow cytometry assay to detect ADAs against CAR T cells using a commercially available molecule to mimic the presence of ADAs. In parallel, we optimized a killing assay to prove the functional activity of the CAR T cells and demonstrated that the ADA-like molecule was able to interfere with the cytotoxic activity of the CAR T cells, highlighting the importance of an extensive characterization of the immune response.

This ADA-like molecule was detected and measured in a dose-dependent manner, showing that flow cytometry was sensitive enough to detect different concentrations of ADAs. A cut point was also calculated that enabled further characterization of the assay, by providing a limit of detection based on the general naïve population binding response.

The flow cytometry assay developed in the current publication has demonstrated the ability to establish key elements (positive control, functional assay, panel decision) and endpoints (cut point) needed to characterize the immune response that can help define and shorten future analysis needed for new CAR T cell products. This ADA by flow assay approach is currently in use at Labcorp for different CAR T cell products, demonstrating its suitability for routine ADA assessment within a regulatory context.

## Supplementary Material

vlag011_Supplementary_Data

## Data Availability

The data that support the findings of this study are available from the corresponding author upon reasonable request. All relevant datasets generated and/or analyzed during the current study have been included in the manuscript and supplementary materials.
