# Supplementary material for: Development of a flow cytometry method to measure antidrug antibodies against CAR T cells
Source: Immunohorizons. 2026 Apr 25;10(4):vlag011. doi: 10.1093/immhor/vlag011 (PMC13109846; doi:10.1093/immhor/vlag011)
Supplement: vlag011_Supplementary_Data [file vlag011_supplementary_data.docx]

**SUPPLEMENTARY INFORMATION**

**Figure S1:** ROR1 CD4 (Panel A) and CD8 (Panel B) CAR T cells were incubated with an IgG_1_ Fc isotype (100µg/mL) as a negative control. The binding of the isotype was then measured in all ROR1 CD4 or CD8 CAR T cells in a histogram using the MFI value of the detection antibody. This value was considered background signal. It was also used to set up a cutline to determine the percentage of ADA^+^ cells, however only the MFI of the detection antibody measured in all ROR1 CD4 or CD8 CAR T cells was used as ADA^+^ signal throughout the study.

**Figure S2:** Cytotoxic activity of ROR1 CD4 and CD8 CAR T cells with E:T ratios 1:1 (Panel A) or 1:10 (Panel B) in the presence of different concentrations of ROR1-IgG_1,_ an ADA-like molecule. ROR1 CD4 or CD8 CAR T did not kill Raji cells expressing ROR1 when E:T ratio was 1:10 (Panel B). When E:T ratio was 1:1, target cells showed low viability (Panel A) however this could be due to the low number of cells used (1 = 25,000 cells). A slight increase in the percentage of live Raji cells was detected with increasing concentrations of the ADA-like molecule ROR1-IgG_1_ when these were cocultured with ROR1 CD8 CAR T cells. Raji cells cocultured with ROR1 CD4 CAR T cells showed higher viability compared with a coculture with ROR1 CD8 CAR T cells, and no effect of the ADA-like molecule was observed. Figure shows data representative from one experimental occasion (Total number of experimental occasions n =1).

**FIGURE S1: Negative control for ADA^+^ detection**

**A**

**FIGURE S1: Negative control for ADA+ detection**

**B**

**FIGURE S2: Cytotoxicity assay results with additional E:T ratios**

**A**

**B**
